# Robust PMBM Filter with Unknown Detection Probability Based on Feature Estimation

**DOI:** 10.3390/s22103730

**Published:** 2022-05-13

**Authors:** Yi Wang, Peng Rao, Xin Chen

**Affiliations:** 1 Shanghai Institute of Technical Physics, Chinese Academy of Sciences, Shanghai 200083, China; wangyi@mail.sitp.ac.cn (Y.W.); chenxin@mail.sitp.ac.cn (X.C.); 2 Key Laboratory of Intelligent Infrared Perception, Chinese Academy of Sciences, Shanghai 200083, China; 3 University of Chinese Academy of Sciences, Beijing 100049, China

**Keywords:** PMBM filter, unknown detection probability, feature estimation, inverse gamma-gaussian mixture

## Abstract

This paper provides a solution for multi-target tracking with unknown detection probability. For the standard Poisson Multi-Bernoulli Mixture (PMBM) filter, the detection probability is generally considered a priori. However, affected by sensors, the features used for detection, and other environmental factors, the detection probability is time-varying and unknown in most multi-target tracking scenarios. Therefore, the standard PMBM filter is not feasible in practical scenarios. In order to overcome these practical restrictions, we improve the PMBM filter with unknown detection probability using the feature used for detection. Specifically, the feature is modeled as an inverse gamma distribution and the target kinematic state is modeled as a Gaussian distribution; the feature is integrated into the target kinematic state to iteratively estimate the target detection probability with the motion state. Our experimental results show that the proposed method outperforms the standard PMBM filter and the robust PMBM filter based on Beta distribution in the scenarios with unknown and time-varying detection probability. Further, we apply the proposed filter to a simulated infrared image to confirm the effectiveness and robustness of the filter.

## 1. Introduction

Multi-target tracking (MTT) refers to the process of jointly estimating the state and number of targets based on the observation data obtained by sensors [1]. MMT is a central problem both in military fields and civilian fields such as defense, surveillance, biomedical research, and autonomous driving [1,2,3,4,5]. Two main types of algorithms have been developed over the past few decades: traditional MTT algorithms and MTT algorithms based on random finite set (RFS) [6,7]. The former, which simplifies the MMT to single-target tracking by solving data association problem between targets and observations, mainly includes Multiple Hypotheses Tracking (MHT) [8,9], Joint Probabilistic Data Association (JPDA) [10], etc., while the latter, which has received much attention and extensive research, due to its providing an effective solution to the data association problem, mainly consists of a Probability Hypothesis Density (PHD) [11] filter, Cardinality-PHD (CPHD) [12] filter, Multi-target multi-Bernoulli (MeMBer) [6] filter, Generalized Labeled Multi-Bernoulli (GLMB) [13,14,15] filter, and Poisson Multi-Bernoulli Mixture (PMBM) [16] filter. Among these algorithms, the PMBM filter, which is a convolution of Poisson RFS and multi-Bernoulli mixture (MBM) RFS, is considered a promising tracking algorithm thanks to its high performance and reduced computational cost [2,17].

It is worth mentioning that the standard PMBM filter requires some a priori knowledge, such as the detection probability, and it is usually considered as a fixed value. However, the detection probability is unknown and even time-varying in practical applications; a significant mismatch in detection probability can result in a significant bias or erroneous estimation, which will affect the practicability of the algorithm. In order to make the filter more suitable for practical scenarios, the robust CPHD/PHD filter [18,19], robust multi-Bernoulli filter [20] and the Cardinality-Balance MeMBer filter [21], the GLMB filter with unknown detection probability [22], and the robust PMBM filter [23] have been successively proposed. All of them model the detection probability as a Beta distribution and iteratively estimate the variable with the targets’ state. However, the performance of these filters degrades when the detection probability is low; the performance also suffers when the model initialization parameters are poor.

Target detection probability depends on the sensor, environment and the features used for detection [24,25]. Generally, the signal-to-noise ratio (SNR) and the amplitude are the most widely used features for detection [26,27]. The SNR feature is introduced to PHD/CPHD to estimate the detection probability [25], while [26] introduces the amplitude to the PHD/CPHD filter to improve filters’ performance, and [28] introduces the amplitude to multi-sensor MeMBer/CPHD to estimate the detection probability; simulation results show that the performance of the filters is improved thanks to the above.

Inspired by [25,26,28], we improve the PMBM filter with unknown detection probability using the feature used for detection. Firstly, we model the feature as an inverse gamma distribution. Then, the feature is integrated into the target kinematic state which is modeled as a Gaussian distribution. In particular, the feature is independent with the kinematic state of targets due to the feature being a relatively stable variable which will not change significantly with the small change in target location. Finally, the detection probability and the motion state can be estimated iteratively. Two outcomes of the experiment can be summarized as (a) the tracking performance of the proposed filter is similar to the standard PMBM filter, where the detection probability is exactly known, fixed and high. Whereas in the low probability scenario, the Beta Gaussian Mixture (BGM-PMBM) filter and standard PMBM filter yield slightly higher error than the proposed filter, especially the BGM-PMBM filter. (b) when the detection probability is varying with time, the proposed filter can more accurately estimate the varying detection probability and has better performance. The results presented show the robustness and effectiveness of the proposed filter.

The key contribution of this paper is the derivation of a close-form solution to the robust PMBM filter recursion which can jointly estimate the target state and detection probability. The intent is to exploit the inverse gamma (IG) component to estimate the feature used for detection and thus the detection probability. The algorithm in this paper improves the tracking performance of the target under unknown detection probability, especially when the detection probability is relatively low.

The outline of the remainder of this paper is as follows. Section 2 introduces the necessary background knowledge of the proposed algorithm, including PMBM recursion and inverse gamma (IG) distribution. Section 3 derives the proposed filter and its implementation. Section 4 gives the simulation results with a linear multi-target filtering scenario. A conclusion is provided in Section 5.

## 2. Background

### 2.1. PMBM RFS Density

Let $x_{k,1},\ldots ,x_{k,N\left(k\right)}$ and $z_{k,1},\ldots ,z_{k,M\left(k\right)}$ denote the states of all N(k) targets and all M(k) measurements at time k, respectively. Then we have RFSs $X_{k}=\left\{x_{k,1},\ldots ,x_{k,N\left(k\right)}\right\}\subset X$, $Z_{k}=\left\{z_{k,1},\ldots ,z_{k,M\left(k\right)}\right\}\subset Z$ which denote the multi-target state set and multi-target observation set, respectively. X denotes the state space and Z denotes the observation space.

According to the observation, the given non-empty state set X can be divided into two parts: undetected targets subsets $X^{u}$ and detected targets subsets $X^{d}$. Hence the posterior density can be denoted as follows:

(1) $$f\left(X\right)=\sum_{X^{u}⊎X^{d}=X}f^{P}\left(X^{u}\right)f^{mbm}\left(X^{d}\right),$$

(2) $$f^{P}\left(X^{u}\right)=e^{-\int \mu \left(x\right)dx}\prod_{x\in X^{u}}\mu \left(x\right),$$

(3) $$f^{mbm}\left(X^{d}\right)=\sum_{j\in I}\sum_{{⊎}_{i\in I^{j}}X^{i}=X^{d}}\prod_{i\in I^{j}}\omega^{j,i}f^{j,i}\left(X^{i}\right),$$

where $f^{P}\left(X^{u}\right)$ is the Poisson density, denoting the undetected targets, μ(x) is the intensity function. $f^{mbm}\left(X^{d}\right)$ is a multi-Bernoulli mixture denoting the potential targets which are detected at least once. $\omega^{j,i}$ is the hypothesis weight and $\sum \omega^{j,i}=1$, $f^{j,i}\left(X^{i}\right)$ denotes the ith Bernoulli density in the jth global hypothesis, which is given by

(4) $$f^{j,i}\left(X^{i}\right)=\{\begin{array}{c} 1-r^{j,i}\;\;\;\;\;\;\;\;\;\;X^{i}=\emptyset \\ \;r^{j,i}\;\;p^{j,i}\left(x\right)\;\;\;\;\;\;X^{i}=\left\{x\right\}\;\;\\ 0\;\;\;\;\;\;\;\;\;\;\;\;\;\;\;\;\;\;\;\;otherwise\;\;\; \end{array},$$

with $r^{j,i}$ denoting the existence probability and $p^{j,i}\left(x\right)$ denoting the single target density.

### 2.2. PMBM Filter

For the standard PMBM filter, the recursive processes are summarized as follows [16].

Prediction step: Suppose the intensity of Poisson RFS $\mu_{k-1}\left(x\right)$ and the MBM RFS ${\left\{\omega_{k-1}^{j,i},r_{k-1}^{j,i},p_{k-1}^{j,i}\left(x\right)\right\}}_{k-1}^{j,i}$ are given at time k−1. The prediction step can be expressed by

(a) For Poisson Component

(5) $$\mu_{k|k-1}\left(x\right)=\gamma_{k}\left(x\right)+\int f_{k|k-1}\left(x|\zeta \right)P_{S,k}\left(\zeta \right)\mu_{k-1}\left(\zeta \right)d\zeta$$

where $\gamma_{k}\left(x\right)$ is the intensity of the new born targets, $\mu_{k|k-1}\left(x\right)$ denotes the predicted intensity. $f_{k|k-1}\left(x|\zeta \right)$ and $P_{S,k}\left(\zeta \right)$ are the transition density of state x given ζ and survival probability given ζ, respectively.

(b) For MBM Component

(6) $$\omega_{k|k-1}{}^{j,i}=\omega_{k-1}{}^{j,i}$$

(7) $$\;r_{k|k-1}{}^{j,i}=r_{k-1}{}^{j,i}\int P_{S,k}\left(\zeta \right)p_{k-1}^{j,i}\left(\zeta \right)d\zeta$$

(8) $$p_{k|k-1}^{j,i}\left(x_{k}\right)\propto \int f_{k|k-1}\left(x|\zeta \right)P_{S,k}\left(\zeta \right)p_{k-1}^{j,i}\left(\zeta \right)d\zeta$$

Update step: Given the predicted PMBM filter with parameters $\mu_{k|k-1}\left(x\right)$ and ${\left\{\omega_{k|k-1}^{j,i},r_{k|k-1}^{j,i},p_{k|k-1}^{j,i}\left(x,a\right)\right\}}_{k-1}^{j,i}$ at time k, the update step can be obtained based on the observations $Z_{k}$.

(a) For Poisson Component

(9) $$\mu_{k|k}\left(x\right)=\left(1-P_{D}\right)\mu_{k|k-1}\left(x\right)$$

where $P_{D}$ denotes the detection probability.

(b) For MBM Component: The update step of detected targets can be divided into two types.

Update for the targets detected for the first time:

(10) $$r_{k}^{P}=e_{k}\left(z\right)/\rho_{k}^{P}$$

(11) $$p_{k}^{P}(x|z)=P_{D}g_{k}(z|x)\mu_{k|k-1}\left(x\right)/e_{k}\left(z\right)$$

where

(12) $$\rho_{k}^{P}=e_{k}\left(z\right)+c\left(z\right)$$

(13) $$e_{k}\left(z\right)=\int P_{D}g_{k}(z|\zeta )\mu_{k|k-1}\left(\zeta \right)d\zeta$$

where $g_{k}(z|x)$ is the likelihood function, and c(z) is the clutter intensity.

Update for the targets detected previously:

(14) $$\omega_{k}^{j,i}=\{\begin{array}{c} \omega_{k|k-1}^{j,i}\times \left(1-r_{k|k-1}^{j,i}+r_{k|k-1}^{j,i}\int p_{k|k-1}^{j,i}\left(\zeta \right)\left(1-P_{D}\right)d\zeta \right)\;\;Z_{k}=\emptyset \\ \omega_{k|k-1}^{j,i}r_{k|k-1}^{j,i}\times \int P_{D}p_{k|k-1}^{j,i}\left(\zeta \right)g_{k}(z|\zeta )d\zeta \;\;\;\;\;\;\;\;\;\;\;\;\;\;\;\;\;\;\;\;\;\;\;\;\;\;\;\;\;\;Z_{k}\neq \emptyset \end{array}$$

(15) $$r_{k}^{j,i}=\{\begin{array}{c} \frac{\;r_{k|k-1}^{j,i}\int p_{k|k-1}^{j,i}\left(\zeta \right)\left(1-P_{D}\right)d\zeta }{1-r_{k|k-1}^{j,i}+r_{k|k-1}^{j,i}\int p_{k|k-1}^{j,i}\left(\zeta \right)\left(1-P_{D}\right)d\zeta }\;\;\;\;\;\;\;\;Z_{k}=\emptyset \\ \;\;\;\;\;\;\;\;\;\;\;\;\;\;\;\;\;\;\;\;\;\;\;\;1\;\;\;\;\;\;\;\;\;\;\;\;\;\;\;\;\;\;\;\;\;\;\;\;\;\;\;\;\;\;\;\;\;\;Z_{k}\neq \emptyset \end{array}$$

(16) $$p_{k}^{j,i}\left(x|z\right)=\{\begin{array}{c} \frac{\left(1-P_{D}\right)p_{k|k-1}^{j,i}\left(x\right)}{\int \int p_{k|k-1}^{j,i}\left(\zeta \right)\left(1-P_{D}\right)d\zeta }\;Z_{k}=\emptyset \;\;\\ \frac{P_{D}g_{k}(z|x)p_{k|k-1}^{j,i}\left(x\right)}{\int P_{D}p_{k|k-1}^{j,i}\left(\zeta \right)g_{k}(z|\zeta )d\zeta }Z_{k}\neq \emptyset \end{array}$$

After the update, we get all possible new single-target hypotheses, we have to go through all possible data association hypotheses to construct the global hypotheses. In order to reduce the cost, a Gibbs sampler [29] or Murty’s [14,16] algorithm can be employed to prune the number of the hypotheses to improve the computation efficiency. In this work, we use the Gibbs sampler due to the lower computation complexity [30]. The detail implementation can be found in [29,31].

### 2.3. Gamma Distribution and Inverse Gamma Distribution

The probability density of the inverse Gamma distribution for non-negative variable a can be denoted as [25]

(17) $$IG\left(a;s;t\right)=\frac{t^{s}}{\Gamma \left(s\right)}a^{-s-1}\exp\left(-\frac{t}{a}\right),$$

where shape parameter *s* > 0 and scale parameter t>0. Γ(s) is Gamma function. The mode at which the probability density function is the maximum is t/(s+1). The mean value and variance of the IG distribution is t/(s−1) and $t^{2}/\left[{\left(s-1\right)}^{2}\left(s-2\right)\right]$ respectively.

The probability density of the Gamma distribution for variable a is as follows

(18) $$G\left(a;s;t\right)=\frac{t^{s}}{\Gamma \left(s\right)}a^{s-1}\exp\left(-ta\right)$$

where shape parameter s>0 and scale parameter t>0. The mode and the mean value are (s−1)/t and s/t respectively.

## 3. The Proposed Robust Filter with Unknown Detection Probability

In this section, the specific implementation of the robust PMBM filter based on inverse gamma Gaussian mixture (IGGM) distribution is introduced.

### 3.1. Target State Model and Observation Model

Similar to [23,25], the feature denoted by a is augmented to x which denotes the kinematic state of a single target and consists of positions and velocities; let $\hat{x}$ expresses the new state of a single target, i.e., $\hat{x}=\left(x,a\right)$. The variable a denotes the SNR throughout in this paper. The detection probability and survival probability at time k can be expressed as

(19) $$P_{D,k}\left(\hat{x}\right)=P_{D,k}\left(a\right),$$

(20) $$P_{s,k}\left(\hat{x}\right)=P_{s,k}\left(x\right)$$

The kinematic state and the feature are modeled as Gaussian distribution and inverse gamma distribution, respectively, so the target density at time k can be denoted by the IGGM as

(21) $$f_{k}\left(x,a\right)=\overset{J_{k}}{\underset{i=1}{\sum }}\omega_{k}^{i}N\left(x;m_{k}^{i};P_{k}^{i}\right)IG\left(a;s_{k}^{i};t_{k}^{i}\right)$$

where $J_{k}$ is the number of the IGGM components at time k, and $\omega_{k}^{i}$ is the weight of the ith IGGM component. m and P are the mean and covariance of Gaussian density. The Markov transition density can be expressed as

(22) $$f_{k|k-1}\left(\hat{x}|\hat{\zeta }\right)=f_{k|k-1}\left(x,a|\zeta ,\alpha \right)=f_{k|k-1}\left(x|\zeta \right)f_{k|k-1}\left(a|\alpha \right),$$

(23) $$f_{k|k-1}\left(x|\zeta \right)=N\left(x;F_{k-1}x_{k-1};Q_{k-1}\right),$$

(24) $$f_{k|k-1}\left(a|\alpha \right)=IG\left(a;s_{k|k-1};t_{k|k-1}\right),$$

(25) $$s_{k|k-1}=k_{s}s_{k-1},0<k_{s}<1,$$

(26) $$t_{k|k-1}=\frac{t_{k-1}}{s_{k-1}-1}\left(k_{s}s_{k-1}-1\right),$$

$F_{k-1}$ and $Q_{k-1}$ denote the state transition matrix and process noise covariance.

Similarly, the observation state is also augmented; the new observation state is expressed as $\hat{z}=\left(z,h\right)$ where z and h are the observation of position and the feature, respectively. The likelihood function of the augmented state at time k can be expressed

(27) $$g_{k}\left(\hat{z}|\hat{x}\right)=g_{k}\left(z,h|x,a\right)=g_{k}\left(z|x\right)g_{k}\left(h|a\right)=N\left(z;H_{k}x;R_{k}\right)G\left(h;\xi ;\frac{\xi }{a}\right),$$

$H_{k}$ and $R_{k}$ represent the observation matrix and observation noise covariance, respectively. The likelihood of feature is gamma distribution which ensures the conjugation of the PMBM filter.

The update of the IG component can be calculated as follows [25].

(28) $$\begin{array}{ll} IG\left(a;s_{k|k-1};t_{k|k-1}\right)g_{k}\left(h|a\right) & \\ & \;=\frac{t_{k|k-1}{}^{s_{k|k-1}}}{\Gamma \left(s_{k|k-1}\right)}a^{-s_{k|k-1}-1}\exp\left(-\frac{t_{k|k-1}}{a}\right)\times G\left(h;\xi_{k};\frac{\xi_{k}}{a}\right)\\ & \;=A\cdot IG\left(a;s_{k};t_{k}\right) \end{array}$$

(29) $$A=\frac{t_{k|k-1}{}^{s_{k|k-1}}}{\Gamma \left(s_{k|k-1}\right)}\frac{\xi^{\xi }}{\Gamma \left(\xi \right)}\frac{\Gamma \left(s_{k}\right)}{t_{k}{}^{s_{k}}}h^{\xi -1}$$

(30) $$s_{k}=s_{k|k-1}+\xi$$

(31) $$t_{k}=t_{k|k-1}+h\xi$$

### 3.2. The Implementtation of Proposed Algorithm

Similar to the standard PMBM filter, the proposed algorithm can be divided into Poisson components and MBM components which denote undetected targets and potentially detected targets, respectively. Besides, the iterative recursion of the Gaussian component is similar to Kalman filter (KF).

Prediction step: Given the intensity of Poisson RFS $\mu_{k-1}\left(x,a\right)$ and MBM RFS ${\left\{\omega_{k-1}^{j,i},r_{k-1}^{j,i},p_{k-1}^{j,i}\left(x,a\right)\right\}}_{k-1}^{j,i}$ at time k−1. The prediction step can be expressed by

(a) For Poisson Component

(32) $$\mu_{k|k-1}\left(x,a\right)=\gamma_{k}\left(x_{k},a_{k}\right)+\overset{J_{k-1}^{u}}{\underset{i=1}{\sum }}\omega_{k|k-1}^{i,u}N\left(x;m_{k|k-1}^{i,u};P_{k|k-1}^{i,u}\right)IG\left(a;s_{k|k-1}^{i,u};t_{k|k-1}^{i,u}\right)$$

where $s_{k|k-1}^{i,u}$ and $t_{k|k-1}^{i,u}$ can be obtained according to (25), (26). $\gamma_{k}\left(x_{k},a_{k}\right)$ is also the IGGM form.

(33) $$\omega_{k|k-1}^{i,u}=p_{S,k}\omega_{k-1}^{i,u},$$

(34) $$m_{k|k-1}^{i,u}=F_{k-1}m_{k-1}^{i,u},$$

(35) $$P_{k|k-1}^{i,u}=Q_{k-1}+F_{k-1}P_{k-1}^{i,u}F_{k-1}^{T}.$$

(b) For MBM Component

(36) $$\omega_{k|k-1}^{j,i}=\omega_{k-1}^{j,i},$$

(37) $$r_{k|k-1}^{j,i}=p_{S,k}r_{k-1}^{j,i},$$

(38) $$p_{k|k-1}^{j,i}\left(x,a\right)=N\left(x;m_{k|k-1}^{j,i};P_{k|k-1}^{j,i}\right)IG\left(a;s_{k|k-1}^{j,i};t_{k|k-1}^{j,i}\right),$$

where

(39) $$m_{k|k-1}^{j,i}=F_{k-1}m_{k-1}^{j,i},$$

(40) $$P_{k|k-1}^{j,i}=Q_{k-1}+F_{k-1}P_{k-1}^{j,i}F_{k-1}^{T},$$

$s_{k|k-1}^{i,u}$ and $t_{k|k-1}^{i,u}$ can be obtained according to (25) and (26).

Update step: Suppose the predicted intensity of Poisson RFS $\mu_{k|k-1}\left(x,a\right)$ and MBM RFS ${\left\{\omega_{k|k-1}^{j,i},r_{k|k-1}^{j,i},p_{k|k-1}^{j,i}\left(x,a\right)\right\}}_{k-1}^{j,i}$ are given at time k, the update step can be expressed by

(a) For Poisson Component

(41) $$\mu_{k}\left(x,a\right)=\left(1-P_{D,k}\left(a_{k|k-1}^{i,u}\right)\right)\mu_{k|k-1}\left(x,a\right).$$

(b) For MBM Component: The update step of detected targets can be divided into two types

Update for the targets detected for the first time

(42) $$r_{k}^{P}=e_{k}\left(z,h\right)/\rho_{k}^{P}\left(z,h\right),$$

(43) $$p_{k}^{P}\left(x,a|z,h\right)=\frac{1}{e_{k}\left(z,h\right)}\times \sum_{i=1}^{J_{k|k-1}^{u}}P_{D,k}\left(a_{k|k-1}^{i,u}\right)\omega_{k,2}^{i,u}A_{k,2}\left(h\right)q_{k,2}\left(z\right)\times N\left(x;m_{k,2}^{i,u};P_{k,2}^{i,u}\right)IG\left(a;s_{k,2}^{i,u};t_{k,2}^{i,u}\right)$$

where $J_{k|k-1}^{u}$=$J_{k-1}^{u}+\left|\gamma_{k}\right|,$ $\omega_{k,2}^{i,u}=\omega_{k|k-1}^{i,u},s_{k,2}^{i,u}=s_{k|k-1}^{i,u}+\xi ,t_{k,2}^{i,u}=t_{k|k-1}^{i,u}+\xi h$.

(44) $$\rho_{k}^{P}\left(z,h\right)=e_{k}\left(z,h\right)+\kappa_{k}\left(z,h\right),$$

(45) $$\kappa_{k}\left(z,h\right)=\lambda_{k}c\left(z\right)IG\left(a;s_{k,2}^{\kappa };t_{k,2}^{\kappa }\right)g\left(h|a\right)=\lambda_{k}c\left(z\right)\frac{{\left(t_{k,2}^{\kappa }\right)}^{s_{k,2}^{\kappa }}}{\Gamma \left(s_{k,2}^{\kappa }\right)}\frac{\xi^{\xi }}{\Gamma \left(\xi \right)}\frac{\Gamma \left(s_{k,2}^{\kappa }+\xi \right)}{{\left(t_{k,2}^{\kappa }+h\xi \right)}^{\left(s_{k,2}^{\kappa }+\xi \right)}}h^{\xi -1},$$

(46) $$e_{k}\left(z,h\right)=\sum_{l=1}^{J_{k|k-1}^{u}}P_{D,k}\left(a_{k|k-1}^{l,u}\right)\omega_{k|k-1}^{l,u}q_{k,2}\left(z\right)A_{k,2}\left(h\right),$$

(47) $$A_{k,2}\left(h\right)=\frac{{\left(t_{k|k-1}^{i,u}\right)}^{s_{k|k-1}^{i,u}}}{\Gamma \left(s_{k|k-1}^{i,u}\right)}\frac{\xi^{\xi }}{\Gamma \left(\xi \right)}\frac{\Gamma \left(s_{k|k-1}^{i,u}+\xi \right)}{{\left(t_{k|k-1}^{i,u}+h\xi \right)}^{\left(s_{k|k-1}^{i,u}+\xi \right)}}h^{\xi -1},$$

(48) $$q_{k,2}\left(z\right)=N\left(z;H_{k}m_{k|k-1}^{i,u};H_{k}P_{k|k-1}^{i,u}H_{k}^{T}+R_{k}\right),$$

(49) $$m_{k,2}^{i,u}=m_{k|k-1}^{i,u}+K\left(z-H_{k}m_{k|k-1}^{i,u}\right),$$

(50) $$P_{k,2}^{i,u}=\left(I-KH_{k}P_{k|k-1}^{i,u}\right),$$

(51) $$K=P_{k|k-1}^{i,u}H_{k}^{T}{\left(H_{k}P_{k|k-1}^{i,u}H_{k}^{T}+R_{k}\right)}^{-1},$$

where $\kappa_{k}\left(z,h\right)$ is the clutter intensity.

Update for the targets detected previously

(52) $$\omega =\{\begin{array}{c} \omega_{k|k-1}^{j,i}\times (1-r_{k|k-1}^{j,i}+r_{k|k-1}^{j,i}\left(1-P_{D,k}\left(a_{k|k-1}^{j,i}\right)\right)\;Z_{k}=\emptyset \\ \omega_{k|k-1}^{j,i}r_{k|k-1}^{j,i}P_{D,k}\left(a_{k|k-1}^{j,i}\right)q_{k,4}\left(z\right)A_{k,4}\left(h\right)\;\;\;\;\;\;\;\;\;\;\;\;\;\;\;\;Z_{k}\neq \emptyset \end{array},$$

(53) $$r=\{\begin{array}{c} \frac{r_{k|k-1}^{j,i}\left(1-P_{D,k}\left(a_{k|k-1}^{j,i}\right)\right)}{1-r_{k|k-1}^{j,i}+r_{k|k-1}^{j,i}\left(1-P_{D,k}\left(a_{k|k-1}^{j,i}\right)\right)}\;\;\;\;\;\;\;\;Z_{k}=\emptyset \\ \;\;\;\;\;\;\;\;\;\;\;\;\;\;\;\;\;\;\;1\;\;\;\;\;\;\;\;\;\;\;\;\;\;\;\;\;\;\;\;\;\;\;\;\;\;\;\;\;\;\;\;\;Z_{k}\neq \emptyset \end{array}$$

(54) $$p=\{\begin{array}{c} N\left(x;m_{k,3}^{j,i};P_{k,3}^{j,i}\right)IG\left(a;s_{k,3}^{j,i};t_{k,3}^{j,i}\right)\;\;\;Z_{k}=\emptyset \;\;\\ N\left(x;m_{k,4}^{j,i};P_{k,4}^{j,i}\right)IG\left(a;s_{k,4}^{j,i};t_{k,4}^{j,i}\right)\;\;\;Z_{k}\neq \emptyset \end{array},$$

where $m_{k,3}^{j,i}=m_{k|k-1}^{j,i}$, $P_{k,3}^{j,i}=P_{k|k-1}^{j,i}$,$s_{k,3}^{j,i}=s_{k|k-1}^{j,i}$,$t_{k,3}^{j,i}=t_{k|k-1}^{j,i}$, $s_{k,4}^{j,i}=s_{k|k-1}^{j,i}+\xi$, $t_{k,4}^{j,i}=t_{k|k-1}^{j,i}+\xi h$.

(55) $$q_{k,4}\left(z\right)=N\left(z;H_{k}m_{k|k-1}^{j,i};H_{k}P_{k|k-1}^{j,i}H_{k}^{T}+R_{k}\right),$$

(56) $$A_{k,4}\left(h\right)=\frac{{\left(t_{k|k-1}^{j,i}\right)}^{s_{k|k-1}^{j,i}}}{\Gamma \left(s_{k|k-1}^{j,i}\right)}\frac{\xi^{\xi }}{\Gamma \left(\xi \right)}\frac{\Gamma \left(s_{k|k-1}^{j,i}+\xi \right)}{{\left(t_{k|k-1}^{j,i}+h\xi \right)}^{\left(s_{k|k-1}^{j,i}+\xi \right)}}h^{\xi -1},$$

(57) $$m_{k,4}^{j,i}=m_{k|k-1}^{j,i}+K\left(z-H_{k}m_{k|k-1}^{j,i}\right),$$

(58) $$P_{k,4}^{j,i}=\left(I-KH_{k}P_{k|k-1}^{j,i}\right),$$

(59) $$K=P_{k|k-1}^{j,i}H_{k}^{T}{\left(H_{k}P_{k|k-1}^{j,i}H_{k}^{T}+R_{k}\right)}^{-1}.$$

The feature can be extracted by

(60) $$a_{k|k-1}^{j,i}=\frac{t_{k|k-1}^{j,i}}{s_{k|k-1}^{j,i}-1}.$$

Then, the detection probability can be obtained by

(61) $$P_{D,k}\left(a\right)=\{\begin{array}{c} \varepsilon_{1}\cdot \left(\exp\left(\frac{a-SNR_{th}}{\delta_{1}}\right)-\varepsilon_{2}\right)\;\;\;\;\;\;\;\;\;\;\;\;a<SNR_{th}\\ \;\varepsilon_{1}\cdot \left(2-\exp\left(-\frac{a-SNR_{th}}{\delta_{2}}\right)-\varepsilon_{2}\right)\;\;a\geq SNR_{th}\; \end{array}$$

Algorithm 1 gives the pseudo-code of the proposed algorithm.

**Algorithm 1** Description of the proposed robust filter
**Input:**

$\;\mu_{k-1}\left(\hat{x}\right)$


$,\;{\left\{\omega_{k-1}^{j,i},r_{k-1}^{j,i},p_{k-1}^{j,i}\left(\hat{x}\right)\right\}}_{k-1}^{j,i}$


$,\;\gamma_{k}$


$\left(\hat{x}\right)$


$,\;{\hat{z}}_{k}$


**Output:**

$\;\mu_{k}\left(\hat{x}\right)$


$,\;{\left\{\omega_{k}^{j,i},r_{k}^{j,i},p_{k}^{j,i}\left(\hat{x}\right)\right\}}_{k}^{j,i}$


$,\;P_{D,k}\;\mathrm{and}\;\mathrm{estimated}\;\mathrm{object}\;\mathrm{states}$


**Prediction process:**
  Poisson process: see Formula (32)  MBM process:   **for**
*j*th global hypothesis **do**        **for**
*i*th Bernoulli in the *j*th global hypothesis **do**     see Formulas (36)–(38)        **end for**   **end for**
**Update process:**
  Poisson process: see Formula (41)  MBM process:       If the target is the first detected     **for** each measurement **do**      Formulas (42) and (43)     **end for**   If the target detected before          **for**
*i*th Bernoulli in the *j*th global hypothesis **do**             Formulas (52)–(54)     **end for****Construct global hypothesis:** Gibbs sampler
**Estimate target state and detection probability**

**Pruning and Merging**


### 3.3. The Computation Complexity of the Proposed Algorithm

Before discussing the computation complexity of the proposed algorithm (IGGM-PMBM), we first analyze the computation complexity of Gaussian Mixture-PMBM (GM-PMBM) [32].

Suppose that the number of association hypotheses is $\left|A_{k-1|k-1}\right|$ after the prediction step at time k−1, it takes $N_{k|k-1}^{u}m_{k}+\left|A_{k-1|k-1}\right|N_{k|k-1}m_{k}+\left|A_{k-1|k-1}\right|{\left(m_{k}/N_{k|k-1}\right)}^{N_{k|k-1}}$ steps to calculate the updated PMBM density at time k, where $N_{k|k-1}^{u}$, $N_{k|k-1}$ and $m_{k}$ are the number of the unknown target components, the MB’s components, and the measurements after the prediction step, respectively. Thus, the complexity of the GM-PMBM update is $O(N_{k|k-1}^{u}m_{k}+\left|A_{k-1|k-1}\right|{\left(m_{k}/N_{k|k-1}\right)}^{N_{k|k-1}})$. The detailed calculations can be found in [33]. From the results, it can be seen that the computation complexity of the PMBM filter is related to the number of the unknown target components, the MB’s components, and the measurements. The framework of IGGM-PMBM filter is unchanged relative to the GM-PMBM filter. Thus, the complexity of the IGGM-PMBM filter does not increase relative to the GM-PMBM filter in theory. However, due to the need to propagate an additional function, i.e., inverse gamma distribution, the IGGM-PMBM filter has a slightly higher complexity compared to the GM-PMBM filter. The same analysis applies to BGM-PMBM filter. Besides, this IGGM-PMBM filter does not increase the complexity of the BGM-PMBM filter but improves the performance.

## 4. Simulation Setup and Results

### 4.1. Simulation Scenario 1

**Figure 1:**
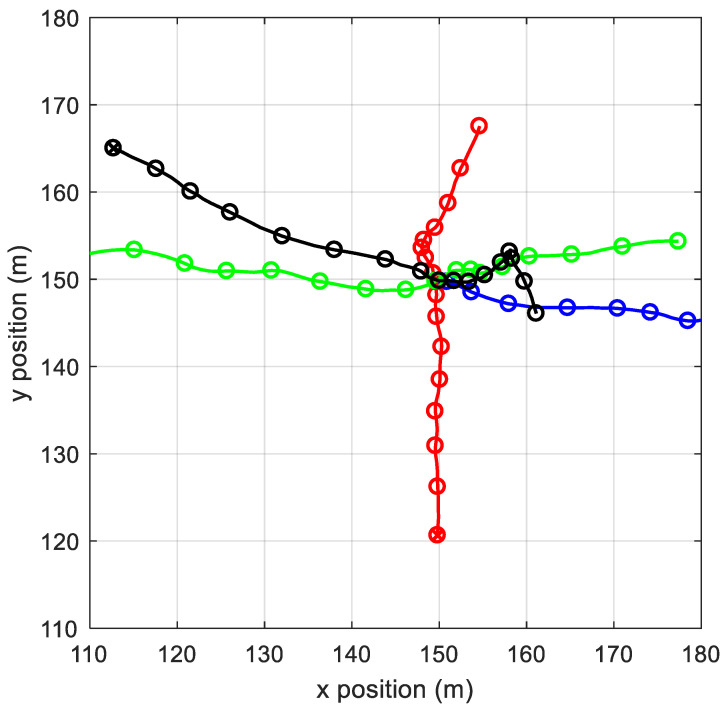
Multi-target scenario. There are four targets, all born at Step1. The blue target dies at Step 40 and the others are alive the entire time.

As a verification for the proposed algorithm, the simulation data in [16] was selected. The area is [110, 180] m × [110, 180] m. There are four targets; Figure 1 is the targets’ real track. The targets are all born at Step 1 and alive all the time, except the blue one which dies at Step 40. Targets kinematic state include the position and velocity $x=\left(p_{x};v_{x};p_{y};v_{y}\right)$ and each observation is a vector of position $z=\left(z_{x};z_{y}\right)$. The parameters used in (23) and (27) are given as

(62) $$F=I_{2}⊗\left(\begin{array}{c} 1\;\;\;T\\ 0\;\;\;1 \end{array}\right),Q=qI_{2}⊗\left(\begin{array}{c} T^{3}/3\;\;\;T^{2}/2\\ T^{2}/2\;\;\;\;\;T\;\;\; \end{array}\right)\;H=I_{2}⊗\left(1\;0\right),R=I_{2}$$

where ⊗ is the Kronecker product, T = 1 is the sampling period, and q=0.01. In formula (25), which denotes the prediction step of IG distribution, $k_{s}$ is set as 0.9. The parameter ξ in likelihood function $g_{k}\left(h|a\right)$ for feature d is 10. In formula (61), we set $\delta_{1}=4$, $\delta_{2}=2$, and $\varepsilon_{1}={\left(2-\exp\left(-SNR_{th}/\delta_{1}\right)\right)}^{-1}$, $\varepsilon_{2}=\exp\left(-SNR_{th}/\delta_{1}\right)$. In the simulation, we let the survival probability be 0.99, i.e., $p_{S,k}=0.99$. The parameters used in the pruning and merging processes are the same as in [16,25]. The proposed algorithm was compared with the GM-PMBM filter and the BGM-PMBM filter which can also estimate the detection probability online. The generalized optimal sub-pattern (GOSPA) [34] assignment metric with parameters α=2 is employed to assess the performance of filters. The GOSPA is defined as

(63) $$d_{p}^{\left(c,2\right)}\left(X,Y\right)≜{\left[\underset{\gamma \in \Gamma^{\left(\left|X\right|,\left|Y\right|\right)}}{\min}\left(\underset{\left(i,j\right)\in \gamma }{\sum }d{\left(x_{i},y_{i}\right)}^{p}+\frac{c^{p}}{2}\left(\left|X\right|+\left|Y\right|-2\left|\gamma \right|\right)\right)\right]}^{\frac{1}{P}}$$

The $\Gamma^{\left(\left|X\right|,\left|Y\right|\right)}$ denotes the assignment set between {1,2,…|X|} and {1,2,…|Y|}. In this situation, $c_{l}^{2}\left(\cdot ,\cdot \right)=\underset{\left(i,j\right)\in \gamma }{\sum }d{\left(x_{i},y_{j}\right)}^{p}$, $c_{m}^{2}\left(\cdot ,\cdot \right)=\frac{c^{p}\left(\left|X\right|-\left|\gamma \right|\right)}{2}$ and $c_{f}^{2}\left(\cdot ,\cdot \right)=\frac{c^{p}\left(\left|Y\right|-\left|\gamma \right|\right)}{2}$ denote the location error (LE), miss error (ME), and false error (FE), respectively. Besides c=1, *p*=2.

There are two cases to be considered: fixed detection probability and time-varying probability.

Case 1-Fixed detection probability: In this scenario, the target detection probability is fixed. Targets are born according to a Poisson process with intensity:

(64) $$\gamma_{k}\left(x_{k},a_{k}\right)=\overset{4}{\underset{i=1}{\sum }}\omega_{\gamma }N\left(x;m_{\gamma }^{i};P_{\gamma }^{i}\right)IG\left(a;s_{\gamma }^{i};t_{\gamma }^{i}\right)$$

where $\omega_{\gamma }=0.03$ and Gaussian density with mean [100;0;100;0], covariance $diag\left(\left[150^{2},1,150^{2},1\right]\right)$. $s_{\gamma }^{i}=51$ and $t_{\gamma }^{i}=500$ denotes the parameters of IG distribution [25], thus the feature used for detection is 10. Clutter is also a Poisson process with intensity $\kappa_{k}\left(z,h\right)=\lambda_{\kappa }IG\left(a;s_{k}^{\kappa };t_{k}^{\kappa }\right)g\left(h|a\right)$, where the clutter rate is $\;\lambda_{\kappa }=10$. The parameters of IG distribution are $s_{k}^{\kappa }=31$ and $t_{k}^{\kappa }=280$. The parameters are summarized in Table 1. The parameters used in the BGM distribution are the same as in [23].

**Figure 2:**
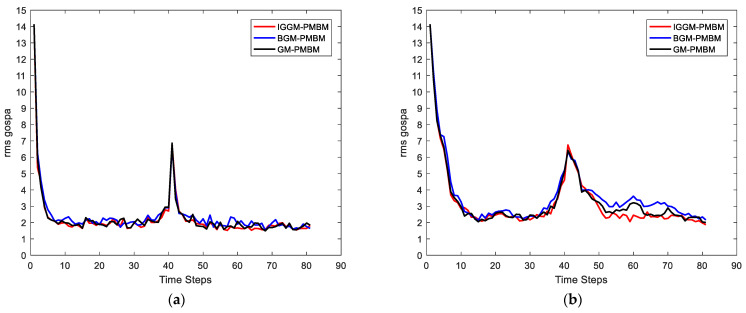
Average of GOSPA distances in 100 Monte Carlo runs. (**a**) $P_{D}=0.94$, $\lambda_{\kappa }=10$; (**b**) $P_{D}=0.68$, $\lambda_{\kappa }=10$.

**Figure 3:**
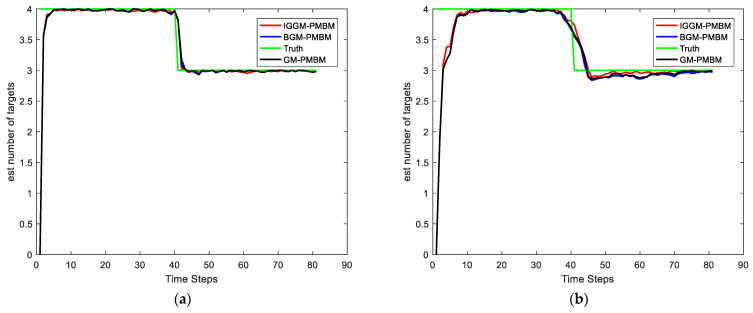
The estimated average number of targets in 100 Monte Carlo runs. (**a**) $P_{D}=0.94$, $\lambda_{\kappa }=10$; (**b**) $P_{D}=0.68$, $\lambda_{\kappa }=10$.

**Figure 4:**
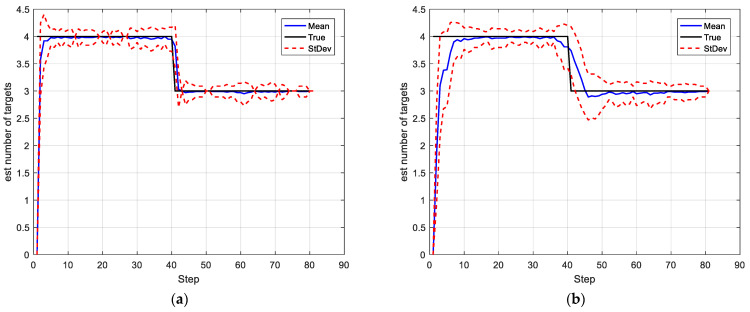
The standard deviation range value and the mean value of the estimated number of targets of IGGM-PMBM filter (**a**) $P_{D}=0.94$, $\lambda_{\kappa }=10$; (**b**) $P_{D}=0.68$, $\lambda_{\kappa }=10$.

**Figure 5:**
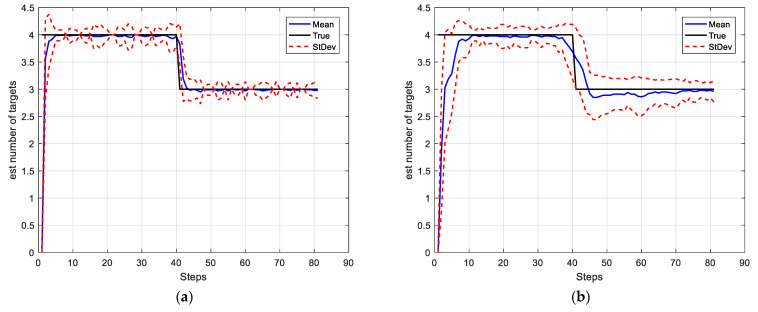
The standard deviation range value and the mean value of the estimated number of targets of BGM-PMBM filter (**a**) $P_{D}=0.94$, $\lambda_{\kappa }=10$; (**b**) $P_{D}=0.68$, $\lambda_{\kappa }=10$.

**Figure 6:**
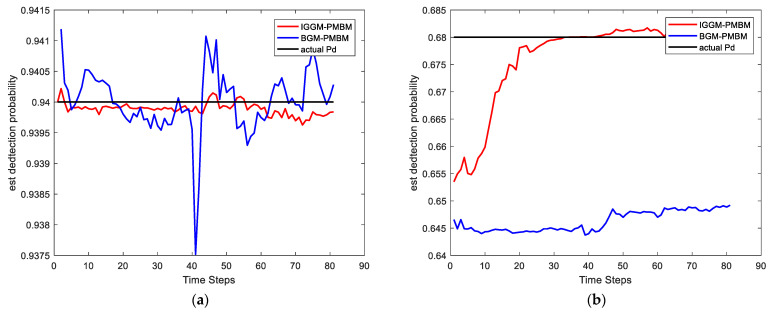
The average of estimated detection probability in 100 Monte Carlo runs. (**a**) $P_{D}=0.94$, $\lambda_{\kappa }=10$; (**b**) $P_{D}=0.68$, $\lambda_{\kappa }=10$.

**Figure 7:**
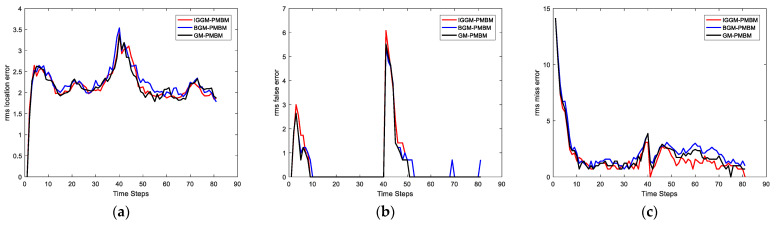
Comparisons of (**a**) location error, (**b**) miss error and (**c**) false error with $P_{D}=0.68$, $\lambda_{\kappa }=10$.

In this simulation, we set the $SNR_{th}$ as two different values: 9 and 5.5, thus the corresponding detection probability is 0.68 and 0.94 respectively. Figure 2 and Figure 3 show the average results, corresponding to the performance metrics on their GOSPA error and the number of targets. In Figure 2, the result shows that the multitarget tracking performance of the proposed IGGM-PMBM filter is similar to the standard GM-PMBM filter with the similar GOSPA distance, where the detection probability is exactly known, fixed and high. Whereas in the low probability scenario, the BGM-PMBM filter and GM-PMBM filter yield slightly higher GOSPA error than IGGM-PMBM, especially the BGM-PMBM. This simulation shows that the proposed IGGM-PMBM filter outperforms the BMG-PMBM filter and GM-PMBM filter with low detection probability. The standard deviation range (StDev) values of estimated number of targets of IGGM-PMBM and BGM-PMBM are shown in Figure 4 and Figure 5. On the other hand, it can be seen in Figure 6 that the estimation of $P_{D}$ of IGGM-PMBM is more accurate. Figure 7 gives the comparisons of LE, ME, and FE when the detection probability is 0.68. The performance comparisons in terms of average GOSPA, LE, ME, and FE with different parameters (detection probabilities and clutter rates) are shown in Table 2. The results show that the performance of the proposed IGGM-PMBM filter is better than the BGM-PMBM filter and the GM-PMBM filter under the same parameters.

The three filters are run separately on an AMD Core 3.20 GHz CPU PC with 16 GB RAM and MATLAB R2021b. The complexity is also illustrated by comparing the computational time. Based on 100 Monte Carlo runs, the average computational times of the three filters are shown in Table 3. It can be seen that the computation complexity of IGGM-PMBM is slightly higher than the GM-PMBM filter in a high detection probability scenario, and with the detection probability decreasing, the IGGM-PMBM filter costs more time to tackle the unknown target detection probability situation. Besides, the IGGM-PMBM filter has almost the same complexity as the BGM-PMBM filter.

**Figure 8:**
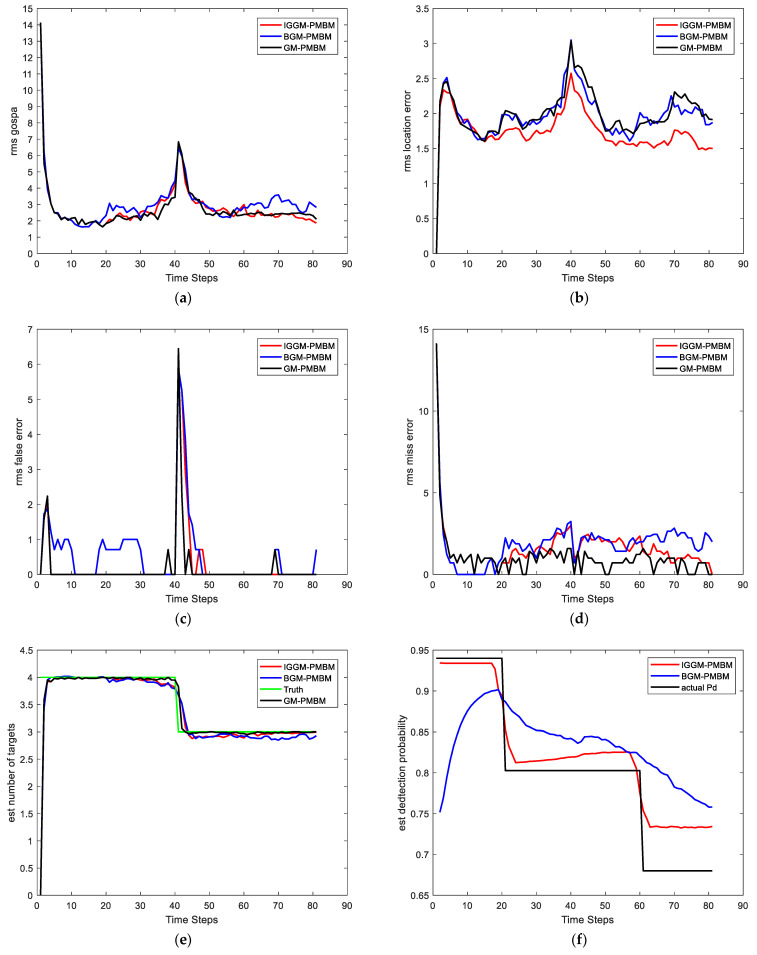
Comparisons of (**a**) GOSPA error, (**b**) Location error, (**c**) False error, (**d**) Miss error, (**e**) the number of targets and (**f**) the estimated $P_{D}$.

**Figure 9:**
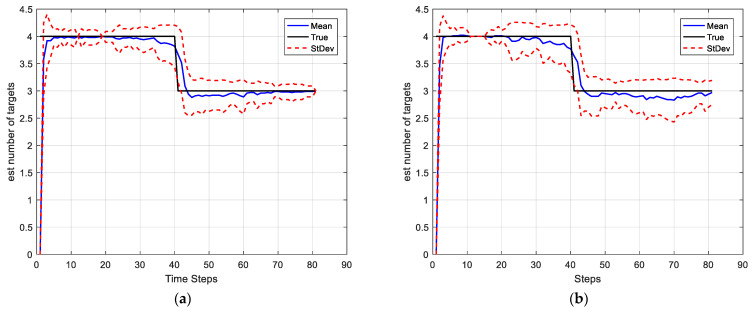
The standard deviation range value and the mean value of the estimated number of targets in the case of detection probability varying. (**a**) IGGM-PMBM filter, (**b**) BGM-PMBM filter.

Case 2-Changing detection probability: In this scenario, the detection probability is varying with time. We set different parameters to express the different feature values for detection as Table 4. The $SNR_{th}$ in this case is 5.5, thus the detection probabilities are 0.94, 0.81, and 0.69 respectively. Other parameters are the same as in Case 1. The average GOSPA error, LE, FE, ME, and cardinality estimate as well as estimate of $P_{D}$ are shown in Figure 8. It can be seen that the estimates of $P_{D}$ of the IGGM-PMBM filter is more accurate than BGM-PMBM filter for each segment. Moreover, the GOSPA errors of the proposed IGGM-PMBM is smaller than that of BGM-PMBM. Figure 9 gives the StDev of the estimated number of targets of the IGGM-PMBM filter and BGM-PMBM filter under the detection probability vary.

### 4.2. Simulation Scenario 2

**Figure 10:**
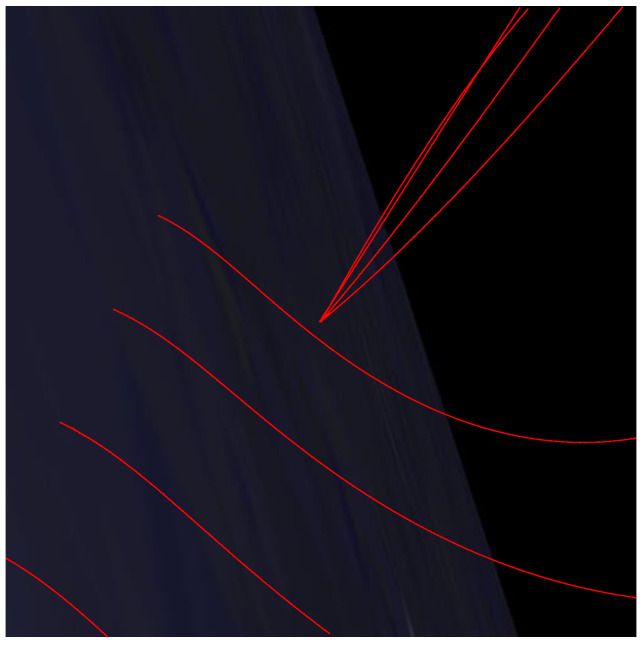
Real trajectory of each target in the infrared image.

**Figure 11:**
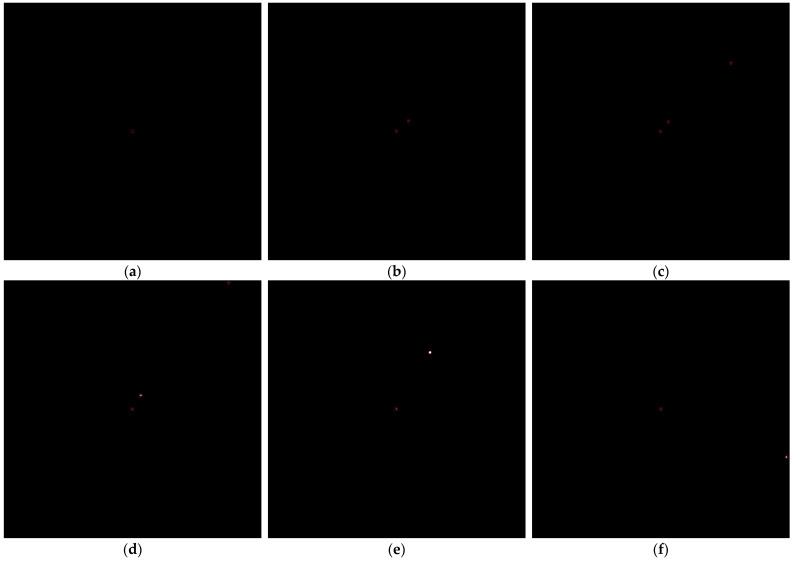

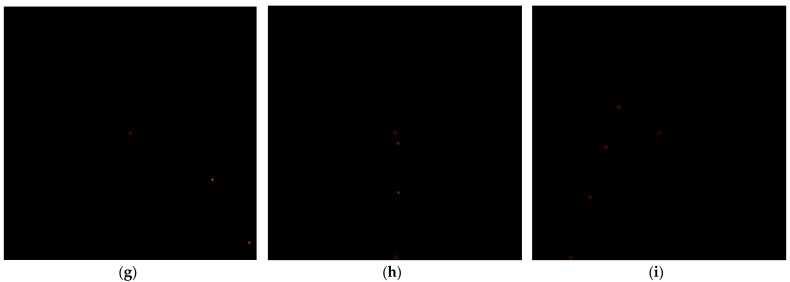
The birth of each target (**a**) First target appears, (**b**) Second target appears, (**c**) Third target appears, (**d**) Fourth target appears, (**e**) Fifth target appears, (**f**) Sixth target appears, (**g**) Seventh target appears, (**h**) Eighth target appears, (**i**) Ninth target appears.

We simulate a long wave infrared image of the movement of space targets using Satellite Tool Kit (STK). The image is 1024 × 1024 pixels, and there are 5400 frames. Figure 10 is the real trajectory of each target in the infrared image. We only track the centroid of the target in this experiment. There are a total of 9 targets, of which target 1 is always in the center of the image. Figure 11 shows the birth state of each target. As shown in Table 5, the birth state of each target including the position and velocity is set according to the target initial states. The covariance is diag([1,1,1,1]).The initial value of the feature for each target is different, and the parameters $s_{\gamma }$ and $t_{\gamma }$ of IG distribution are different at the birth time. The number of frames in which the target is born and the number of frames in which it dies are also given in Table 5. In this scenario, $SNR_{th}$ is 5.5. Other parameters are the same as in simulation scenario1.

**Figure 12:**
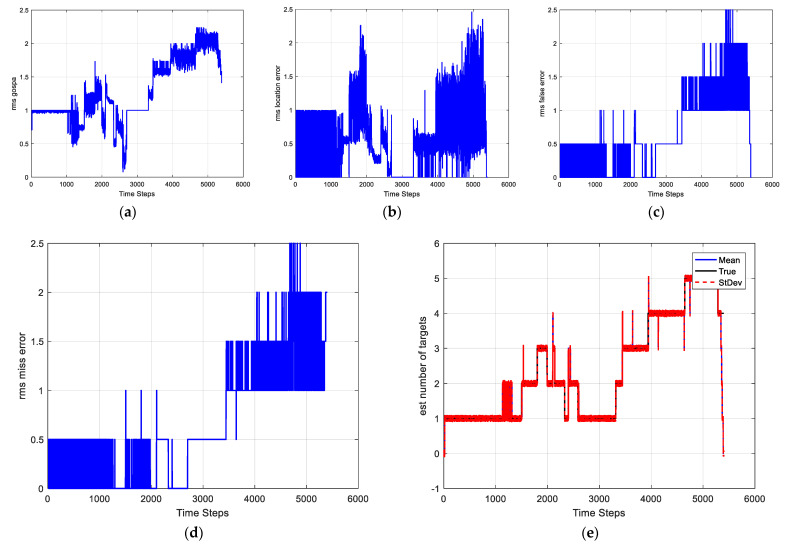
The average GOSPA, LE, FE, ME, and the estimate number of targets of the infrared image (**a**) GOSPA error, (**b**) Location error, (**c**) False error, (**d**) Miss error, (**e**) the number of targets.

Figure 12 gives the performance of the IGGM-PMBM filter used in the infrared image. Obviously, the filter is effective for the infrared image.

## 5. Conclusions

In order to solve the problem of unknown detection probability and improve the accuracy when the low detection probability is low, we propose a novel PMBM filter based on the feature estimation for multi-target tracking. We model the feature as an inverse gamma distribution and integrate it into the kinematic state of targets. The feature can be estimated along with the motion state of the target, so as to further calculate the detection probability. The feature is related to detection and can be actually measured which can improve the accuracy of the detection probability estimation. The simulation results also show that the proposed algorithm can accurately estimate the detection probability and outperforms the GM-PMBM filter and BGM-PMBM filter with slightly lower GOSPA error especially when the detection probability is low. The proposed algorithm was then used in an infrared simulation scenario, which demonstrated the robustness and the effectiveness of the proposed algorithm. With the increase in space targets and development of autonomous driving and other fields, more and more radar data and space-based infrared image data will be obtained. Thus, the verification of the proposed algorithm using real data such as radar data and infrared images data would be a worthwhile subject of future study. Extending the proposed algorithm with unknown information is also a topic worthy of study.

## Figures and Tables

**Table 1:** Parameters of the fixed detection probability.

parameter	$k_{s}$	ξ	$p_{S,k}$	$s_{\gamma }^{i}$	$t_{\gamma }^{i}$	$s_{k}^{\kappa }$	$t_{k}^{\kappa }$	$\lambda_{\kappa }$
value	0.9	10	0.99	51	500	31	280	10

**Table 2:** Root mean square GOSPA and LE, ME, and FE at all time steps with different parameters (*P_D_*, *λ_κ_*).

(*P_D_*, *λ_κ_*)	Proposed IGGM-PMBM				GM-PMBM				BGM-PMBM			
	GOSPA	LE	ME	FE	GOSPA	LE	ME	FE	GOSPA	LE	ME	FE
(0.94, 10)	2.7477	1.7745	1.9277	0.8278	2.7511	1.7780	1.9277	0.8315	2.7592	1.7767	1.9181	0.8819
(0.94, 15)	2.8097	1.7897	1.9798	0.8784	2.7940	1.7876	1.9689	0.8571	2.7963	1.7895	1.9563	0.8889
(0.94, 20)	2.8417	1.7886	2.0154	0.9027	2.8534	1.7985	1.9985	0.9558	2.8538	1.7990	1.9783	0.9969
(0.94, 25)	2.8730	1.7937	2.0215	0.9750	2.9012	1.8063	2.0846	0.8992	2.9098	1.8098	2.0638	0.9655
(0.68, 10)	3.7648	2.2141	2.7499	1.3076	3.8460	2.2045	2.9187	1.1889	3.9832	2.2184	3.0123	1.3676
(0.68, 15)	4.0033	2.2626	3.0358	1.3005	3.9756	2.2422	3.0358	1.2497	4.1448	2.2610	3.1456	1.3494
(0.68, 20)	4.1351	2.2292	3.1407	1.5051	4.2651	2.1971	3.3939	1.3586	4.3428	2.2309	3.4265	1.4635
(0.68, 25)	4.2925	2.2756	3.3370	1.4530	4.3054	2.2758	3.4021	1.3240	4.4715	2.2840	3.5651	1.4380

**Table 3:** Average computational times.

$P_{D}$	Proposed IGGM-PMBM	BGM-PMBM	GM-PMBM
0.94	3.83 s	3.71 s	3.37 s
0.68	6.07 s	5.89 s	5.16 s

**Table 4:** The parameters for different detection probability.

Step	0~20	21~60	61~80
$(s_{\gamma },t_{\gamma }$)	(51, 500)	(51, 385)	(51, 335)

**Table 5:** The target initial states.

Target	State	Feature	$s_{\gamma }$	$t_{\gamma }$	Survival Time (Frame)
1	[512;0;512;0]	6	51	300	[18,5400]
2	[512;0;512;0]	6	51	300	[1501,1991]
3	[512;0;512;0]	8	51	400	[1801,2139]
4	[512;0;512;0]	8.6	51	430	[2101,2329]
5	[512;0;512;0]	9.7	51	485	[2401,2584]
6	[1024;0;701;0]	9.2	41	368	[3318,5400]
7	[1024;0;960;0]	8.8	41	352	[3444,5400]
8	[523;0;1020;0]	6.7	41	268	[3940,5400]
9	[164;0;1024;0]	6	41	240	[4648,5284]

## Data Availability

Not applicable.
